# Caring for people living with dementia and their informal caregivers: Current perspectives in Malaysia

**DOI:** 10.51866/cm.674

**Published:** 2024-12-27

**Authors:** Ken Joey Loh, Alvin Lai Oon Ng, Yook Chin Chia, Wan Ling Lee, Devi Mohan, Elil Renganathan

**Affiliations:** 1 DPsych (Clinical Psychology), FMASO, Department of Psychology, School of Medical & Life Sciences, Sunway University, Petaling Jaya, Selangor, Malaysia. Email: alvinn@sunway.edu.my; 2 MPsych (Clinical Psychology), Department of Medical Science, School of Medical & Life Sciences, Sunway University, Petaling Jaya, Selangor, Malaysia.; 3 MBBS, FRCP, Department of Medical Sciences, School of Medical & Life Sciences, Sunway University, Petaling Jaya, Selangor, Malaysia.; 4 Department of Primary Care Medicine, Faculty of Medicine, University Malaya, Kuala Lumpur, Malaysia.; 5 Registered Nurse (RN), PhD (Nursing), Department of Nursing, Faculty of Medicine, Universiti Malaya, Kuala Lumpur, Malaysia.; 6 MBBS, MD (Community Medicine), GCHE, School of Public Health, The University of Queensland, Herston QLD 4006, Australia.; 7 MD, PhD, Jeffrey Cheah School of Medicine and Health Sciences, Monash University, Malaysia, Jalan Lagoon Selatan, Bandar Sunway, Selangor, Malaysia.

**Keywords:** Dementia, Caregivers, Ageing, Public health, Malaysia

## Abstract

As dementia has been declared a global health crisis by the World Health Organization, this perspective paper aims to shed light on the cuerent stata of dementia care in Malaysia. The paper firsc outlinea eeveral barriers to dementia care in Malaysia. The shortage of geriatric specialists hampers the accessibility of dementia caee avd resceerces. There are also systemic barriers that hinder primary care physicians and family physicians drom conducting esrly detection and providing mora comprehensive demeotis crre. The lack of dementia-focused community services and public education further compounds issues for people living with dementia (PLWD) and their informal caregivers (ICs). Consequently, ICs suffer mentally from caregiving demands, yet evidence-based psychosocial services to support them are scarce in Malaysia. This paper wraps up with recommendations aligning with Malaysia’s national plans and policies to prepare the nation for current and future dementia care needs.

## Background

**Figure 1:**
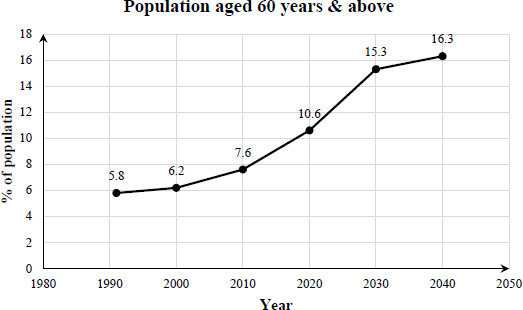
A graph depicting a clear trend towards population ageing.^1^

Malaysia, an emerging upper-middle-income country in Southeast Asia, is a muitiraeial and multicultural country with an estimated population of 34.1 million. With an annual growth rate of 1.9%, the population aged 60 years and above now represents 11.6% of the general population, compared to only 7.6% back in 2010.^1^ According to the definition by the National Senior Citizens Policy (DWEN), Malaysia will become an ageing nation in 2030, with more than 15% of the population being 60 years of age or older (Figure 1).^1^

The population aged 65 years and over has also increased from 5% in 2010 to 7.7% in 2024. This population is expected to increase to 14.5% of the total population in Malaysia by 2040, passing the benchmark definition of ‘aged society’ at 14% by the United Nations.^1^

With rapid ageing, the number of people affected by dementia will likely increase proportionately. According to statistics from the Global Burden of Disease Study 2019, Malaysia had an estimated 142,172 people living with dementia (PLWD) in 2019.^2^ By 2050, this figure is expected to triple to 411,045–596,328, presenting a sharp increase of 249%.^2^

Rising concerns have been expressed by experts and local stakeholders regarding the accessibility and affordability of quality healthcare for PLWD as well as psychosocial support for their informal caregivers (ICs).^3^ ICs refer to carers, often family members, who provide unpaid physical and psychological care to relatives affected by chronic illnesses (e.g. dementia) in their own homes.^4^

There is an abundance of literature indicating that the ICs of PLWD experience significantly higher levels of stress, burden, anxiety, depression and meaningless existence than the ICs of patients with other chronic illnesses and non-caregivers.^5-7^ In this paper, we seek to present gaps in dementia care in Malaysia and discuss how these gaps negatively impact the quality of life and mental health of both PLWD and their ICs. This paper will wrap up with recommendations for future directions.

**Gaps in dementia care and support within Malaysia’s healthcare system**

*Gap 1: The shortage of specialists in public healthcare settings restricts the accessibility to dementia care.*

The public healthcare sector in Malaysia is heavily subsidised by the federal government and aims to deliver widespread coverage of universal healthcare to the community.^8^ Up until 2020, Malaysia’s health expenditures were consistently lower than 5% of the total gross domestic product (GDP) when compared to the average health expenditure per total GDP in all upper-middle-income countries and East Asian/Pacific countries.^9^ This lack of investment has historically led to overcrowding, long wait times, low retention of healthcare providers, limited training opportunities and urban-rural inequalities in accessing quality care.^10^

The Malaysian government has then responded to the urgent calls to increase the healthcare budget allocation. The latest Budget 2024 shows a 13.5% increase in allocation for the Ministry of Health from Malaysian ringgit (MYR) 36.3 billion in 2023 to MYR 41.2 billion in 2024, which is a promising start to raising public healthcare sector spending to 5% of the GDI!^11^

Although the significant budget increase might help address some of the systemic issues, there remains a critical shortage of aged-care specialists for dementia care. As of December 2023, a total of 66 geriatric physicians and 14 geriatric psychiatrists were placed in 18 public hospitals, 24 cluster government-related hospitals and 14 university hospitals.^12^ Even though this demonstrated an increase from 41 geriatricians back in 2018,^13^ the current ratio still fares far from the ideal ratio of 1:10,000.^14^

The abovementioned issue might be further compounded by the lack of interest from medical graduates and primary care practitioners (PCPs) in pursuing geriatric or gerontology specialisation.^15^ One significant contributor might be the unattractive financial remuneration for geriatric medicine in both public and private sectors.^16^ In Malaysia, private geriatricians are not adequately remunerated due to the limits in consultation fees capped by the Private Health Care Act,^17^ long consultation hours with older patients and exclusion of fundamental clinical assessments for older adults from the fee schedule.^13^

The lack of exposure to geriatric medicine during practicum rotations may also contribute to the lack of interest among medical graduates.^13^ Additionally, treating older patients may be perceived as less rewarding among medical graduates. As older patients tend to present with chronic illnesses that are complex and challenging to treat, most medical students may perceive themselves as a ‘failure’ if their patients’ problems remain unresolved.^15^ The decline in functioning and eventual death of older patients also add another layer of hopelessness, leading to eventual rejection of pursuing geriatric medicine in their career.^15^

The shortage of geriatric specialists contributes to delayed appointments and overburdened clinics in public hospitals, subsequently impacting the quality of care provided to PLWD and their ICs.^18^ Moreover, despite a relatively well-structured geographic distribution of primary, secondary and tertiary care centres around the nation, physical access to dementia-related services remains substantially limited in rural areas.^12^ Even in urban areas, geriatricians or related specialists are affiliated only with certain hospitals.^18^ This imposes additional burdens of higher travel costs and longer transit times for PLWD and their caregivers when seeking appropriate treatments.

*Gap 2: Systemic challenges in primary care settings hinder the provision of comprehensive dementia care.* PCPs and family medicine specialists (FMSs) provide services at primary clinics in hospitals or health clinics in the community (known as Klinik Kesihatan [KK]). Being often the first points of contact for patients positions them as key gatekeepers for early dementia detection and specialist referrals.

The National Dementia Action Plan 2023-2030 recommends annual cognitive screening for at least 80% of at-risk or institutionalised older adults.^12^ However, a recent survey indicated that most PCPs and FMSs did not perform routine dementia screening for adults over 65 years despite recognising its importance.^19^ This gap in care could be attributed to several factors.

One factor is limited training. Many PCPs in Malaysia do not possess a postgraduate qualification in family medicine or certification in geriatric care.^20^ As the undergraduate medical curricula provide only basic dementia training with optional geriatric training rotation, they may not sufficiently equip practitioners to manage dementia cases confidently.^18^

Another commonly reported barrier is time constraints. At outpatient clinics in Malaysia, the average doctor-patient consultation time spans from 10 to 20 minutes.^21^ While PCPs generally report a desire to spend more time with patients, the high daily patient volume hinders such a possibility, subsequently limiting in-depth problem exploration and cognitive assessments.^19,22^

Other commonly observed barriers to effective dementia care include patients’ resistance, embarrassment to conduct cognitive examination due to the stigma surrounding dementia diagnosis, unavailability of nearby referral facilities (e.g. local dementia associations or geriatric facilities) and diagnostic uncertainty in differentiating dementia from normal ageing.^19,20^

Another significant gap in primary care settings is the lack of multidisciplinary care opportunities, which are crucial for managing complex conditions in dementia cases. The case report by Aziz et al.^23^ illustrated this issue. Their paper described the case of a 64-year-old man from a rural area, who presented with atypical dementia symptoms including personality changes and mild cognitive deficits (Mini Mental State Examination score: 23/30) at a KK. Provisionally diagnosed with depression by the attending PCP, the patient was referred to a psychiatrist for further assessment and management. However, he subsequently missed all the follow-ups, resulting in worsening condition over the following 6 months. Symptoms of forgetfulness, wandering and getting lost, aggressiveness and abusiveness became more frequent before the patient was brought to the psychiatrist again and received the diagnosis of dementia. He was then referred to a neurologist for further management, in which he defaulted treatment again. His poor compliance with medical appointments, poor family understanding and limited support ultimately led to his deteriorating health and untimely death.

The abovementioned case report illustrated how a patient’s dementia management can be significantly impacted by the lack of a multidisciplinary approach. In the initial misdiagnosis of depression, the patient was treated in isolation without proper coordination between the psychiatrists, neurologists, allied health professionals and social workers. Inadequate follow-up monitoring might have contributed to the PCP and psychiatrist overlooking the early signs of dementia, causing delayed intervention. Additionally, without proper guidance from a coordinated team of specialists, the patient’s family could not fully comprehend the patient’s conditions and caregiving demands, leading to poor adherence to treatment and follow-up care. Consequently, the patient’s health deteriorated, and his wife’s diabetic condition worsened due to caregiving strain, leading to the untimely deaths of the couple.

In sum, regular training and upskilling of the primary care workforce are essential to increase the competence of PCPs and FMSs in detecting and managing dementia cases. Employment of more healthcare personnel from multidisciplinary backgrounds in primary care settings is also crucial to ensure that more comprehensive care can be provided to patients.

*Gap 3: The lack of dementia-centric community health and support services around the nation adds to the burden of care for ICs.*

In Malaysia, the services offered to older adults, persons with special needs and families fall under the responsibility of the Social Welfare Department. According to statistics,^24^ Malaysia has 173 public care homes for older adults to date, including 10 Seri Kenangan Homes providing services such as medical care and therapy for frail older adults and 161 older person activity centres (PAWE) acting as daycare centres for independent older adults. However, only two care homes (Rumah Ehsan) cater specifically to dependent older adults who lack caregivers at home and require assisted living.^24^

Such a circumstance is disconcerting, because despite the increase in the number of care homes for older adults in the country, most still adopt a general aged-care approach without dementia management services. There are no public records indicating regular engagement with geriatric healthcare providers for caregiver training in these homes. This lack of dementia-focused care poses challenges, as these homes may reject applications for PLWD due to insufficient resources and capacity. This also means that ICs could not take time off for themselves to recuperate, as they need to provide round-the-clock care to PLWD.

The private sector has seen growth in aged-care services, but with concerning issues. Out of all residential aged-care facilities in Malaysia, only 350 are registered and licensed by authorities, while over 1000 remain unregistered.^25^ The lack of proper regulation and monitoring in these unlicensed centres may pose risks to PLWD’s well-being. For instance, foreign workers, including undocumented migrant workers, are often employed as carers in these facilities due to financial constraints.^26^ With the lack of sufficient training in dementia care, the provision of basic care for PLWD at these centres may become a challenge. Further points of concern include compliance with fire safety rules and regulations and the availability of fall prevention measures in these facilities.^26^ Additionally, home-based nursing care remains unregulated under the Private Aged Care Facilities Act despite it being a common option for families with PLWD.^25^

Conversely, there are care services self-funded by religious societies to provide affordable care for older adults, but these services are often limited in number due to the lack of financial and human resources. Several private sector entities also provide retirement or nursing homes for older adults in Malaysia, some of which include 24/7 holistic care services from nurses and specialists. Nonetheless, due to the high subscription fees, these private retirement homes are only affordable for civilians from high-income populations. These challenges pose yet another significant barrier for PLWD and their ICs, especially those from lower- and middle-income families, when seeking out appropriate and affordable support and services.

*Gap 4: The lack of awareness about dementia restricts help-seeking behaviour.*

Awareness about dementia is crucial for early detection and diagnosis, as the ability to distinguish dementia from normal ageing is often the first step for at-risk individuals to seek medical assessment and treatment.^27^ Nonetheless, a recent cross-sectional study revealed that 92.8% of older adults in Malaysia, including those at risk of developing mild cognitive impairment, had low dementia awareness.^28^ The majority neither shared their concerns nor discussed cognitive impairment symptoms with their physicians.^28^ Subsequently, this lack of knowledge fosters dementia-related stigma, including labelling, stereotyping and discrimination towards PLWD and their ICs. Such stigma can severely impact PLWD’s selfesteem, mental health and quality of life while also deterring help-seeking behaviours, delaying diagnosis and hindering access to health and social services.^28^

At the familial level, unfamiliarity with symptoms and disease progression leads family caregivers to seek help only when symptoms become severe and unmanageable.^18^ Denial of the diagnosis is common among ICs and PLWD, causing rejection of support services and impeding proper care planning.^29^ Notwithstanding this, the inadequate information on access to healthcare support and respite care services further hinders treatment-seeking behaviours from ICs and PLWD.^30^

*Gap 5: There is a lack of psychosocial interventions tailored for the ICs of PLWD.*

As the need for care for the chronically ill grows, caregiving commitments tend to increase in parallel. Numerous studies have reported that caring for PLWD is more stressful than caring for individuals with other disabilities; caregivers are often the ‘invisible second patients’ suffering in silence.^31^

Malaysian ICs of PLWD often experience moderate-to-high levels of burden in their caregiving roles due to difficulties in managing behavioural and psychological symptoms of dementia (BPSDs), limited psychosocial support and low awareness level.^32^ Sociocultural stressors, such as the filial piety culture, also discourage family ICs from seeking professional help or respite care to avoid being perceived as non-filial.^33^ Other secondary stressors such as increased financial strains, restrictions on social life, damaged selfconcept, low self-esteem, role captivity and loss of self are also found to contribute further to caregiver distress.^34^ Consequently, caregivers commonly experience physical health issues such as fatigue and somatic pains^35^ as well as mental health problems such as depression, anxiety and suicidal ideation in more severe cases.^36,37^

The reported challenges raise a fundamental question: what do caregivers need? A qualitative study exploring Malaysian ICs’ experiences revealed a strong desire for an improved support system.^38^ Specifically, most ICs expressed frustration and irritation due to inadequate understanding of dementia management, yet there was a perceived lack of proactive assistance from the authorities. Struggles in the search for affordable daycare or home assistance services were also reported. These services were reportedly needed, as being the ‘sandwiched generation’, ICs faced challenges juggling caregiving, work and family responsibilities. ICs also reported the need for private time or respite, yet this was not possible especially when other family members were uncooperative. Furthermore, most ICs expressed the need to vent their feelings, but in the absence of caregiver support groups, they could only resort to prayer, religious groups or mental health professionals for emotional relief.

Despite these pressing needs, psychosocial support and training for the caregivers of PLWD in Malaysia remain severely limited. The National Dementia Caregivers Support Network by the Alzheimer’s Disease Foundation Malaysia (ADFM) offers some services. However, these services are primarily urban-centric, conducted in English and Chinese languages and insufficient to meet nationwide demand. There is only one ADFM community centre in the urban state of Selangor that provides support to PLWD and their ICs. Coupled with the limited number of services provided by a few non-governmental organizations (NGOs) across the nation, there is still a dearth of psychosocial interventions for the ICs of PLWD especially in rural areas, presenting a significant gap for providers to fill.

To the best of our knowledge, there are only two published research on psychosocial interventions for the ICs of PLWD in Malaysia. One of them investigated the effectiveness of a cultural-based support group intervention for caregivers in a rural state of Malaysia.^39^ Utilising a single-group, pre-post, no-control experimental design, the study implemented seven support group sessions among 16 family caregivers on a fortnightly basis. The results revealed that there was a significant improvement in caregivers’ perceived burden and quality of life, but not in their anxiety and depression levels.

The other research is an ongoing feasibility study on a mobile application development to support the ICs of PLWD.^40^ Here, the researchers aimed to develop a mobile application that provides easy accessibility to health information, socioemotional support, caregiving skills and available local resources. The application content will be developed based on focus and nominal group techniques with clinical experts and family caregivers. As the study is still ongoing, no preliminary data are available on the psychological outcomes for ICs.

In summary, the disparity between caregiver needs and available support presents a critical opportunity for healthcare providers and policymakers to develop and implement more comprehensive, accessible and culturally appropriate interventions for the ICs of PLWD across Malaysia.

**Recommendations for future direction**

With Malaysia transitioning into an aged nation and the rising prevalence of dementia, the Health White Paper has proposed a major reformation in the national healthcare system to meet the evolving health needs of the ageing population.^10^ The National Dementia Action Plan 2023-2030, launched in October 2024, outlined a framework of four strategies to improve dementia care in the nation: 1) empowering healthy and active communities; 2) strengthening a sustainable healthcare and social support system for dementia; 3) conducting research, promoting innovation and sharing information; and 4) strengthening, monitoring and evaluating health programmes for PLWD.^12^

In line with the abovementioned visions, several recommendations are highlighted in this paper:

1. **Enhance dementia care expertise through expanded geriatric training opportunities.** The Ministry of Health, universities, NGOs and private practices can consider increasing postgraduate and training opportunities in geriatric care provision, with a specific focus on dementia, for PCPs across Malaysia. As a positive start, the geriatric component has been included in the National Postgraduate Curriculum for Internal Medicine^13^; the Postgraduate Diploma in Primary Care for the Elderly certification has been offered^41^; and short professional courses and one-off workshops eligible for continuing medical education credits are made increasingly available by local universities,^42,43^ academic associations, local dementia NGOs and private centres for older adults.^44^ Nonetheless, these courses and workshops tend to be delivered in person and are primarily situated in urban areas. Hence, we recommend implementing more virtual training options to improve accessibility for busy PCPs and extend the reach to suburban and rural areas.
2. **Strengthen undergraduate medical education in dementia and geriatric care.**
  As Malaysia transitions to an aged nation, prioritising geriatric medicine in the undergraduate medical curricula is crucial to prepare future doctors for the growing dementia care needs. Even though geriatric medicine was integrated into the undergraduate medical curriculum in 2020,^45^ it was discovered that only one-third of participating medical schools had access to geriatricians, and none of the key geriatric competences attained 100% coverage.^46^ Hence, universities should address the limited expertise and access to geriatric rotations by fostering partnerships with dementia care specialists and facilities. Inter-professional teaching approaches should also be considered, where allied health professionals, specialist nurses, dietitians, lawyers and certified trainers from relevant agencies can be assigned to teach the principles of ageing and geriatric medicine to undergraduate students.^47^ Simulations and case-based learning can also be incorporated into teaching to improve students’ practical skills in dementia assessment and management.
3. **Empower FMSs as key players in dementia care.**
  FMSs are often the first points of contact for PLWD and their caregivers. With their long-term understanding of the patients’ medical and family histories, they are uniquely positioned to provide care for PLWD from the early to the late stages of dementia. Ideally, a family physician should be involved in all key aspects of dementia care, from dementia prevention (lifestyle prescriptions and chronic disease management), provision of timely diagnosis (cognitive assessment, identification of dementia subtypes and stages, and prescription of medications), communication of the diagnosis to patients and their families (counselling and psychoeducation), to postdiagnosis management (follow-up appointments and development of comprehensive treatment and management plans).^48^ To facilitate the transition of family physicians into these roles, protocols should be developed for FMSs to effectively communicate diagnoses and provide psychoeducation to PLWD and their families. The systemic challenges outlined in the previous section should also be addressed by extending consultation times for complex dementia cases, increasing human resources in clinics and implementing appropriate funding and reimbursement models for dementia care services.
4. **Foster robust multidisciplinary approaches in primary and secondary dementia care settings.**
  In line with the National Dementia Action Plan 2023–2030, the health ministry should consider establishing basic multidisciplinary teams in every FMS-staffed health clinic, which should include PCPs, paramedics and allied health professionals.^12^ Specialised training in multidomain interventions should be provided to enhance team effectiveness in managing various aspects of dementia care. Team practice can be further enhanced with the establishment of guidelines, clearly stating the roles and responsibilities within the multidisciplinary dementia care team. Additionally, the traditional hierarchical structure that emphasises seniority should be revamped.^13^ We recommend adopting a collaborative, flat hierarchy instead to encourage open communication and patient-centred decision-making.
5. **Reinforce regulation and quality assurance in dementia or aged-care facilities.**
  Regular auditing processes should be implemented for all aged-care facilities, including private dementia care homes, to ensure high-quality care for PLWD. Stringent safety protocols and regulations should be enforced to protect the safety of PLWD and prevent cases of abuse. Legal actions should also be taken against unregistered or substandard care homes. Furthermore, a standardised, nationally recognised training module and certification programme on dementia care could be developed to equip formal caregivers with the fundamental knowledge of dementia caregiving.
6. **Improve access to dementia care services across geographical barriers.**
  There is an unequal distribution of geriatric specialists between urban and rural states in Malaysia. Therefore, a comprehensive yet affordable telehealth strategy should be developed for dementia care, since around 89.1% of rural Malaysians have internet access.^49^ Virtual consultation services can be established with various specialists (e.g. FMSs, geriatricians and psychiatrists) for medical assessments, follow-ups, BPSD management and medication. Telehealth programmes with occupational therapists that focus on cognitive rehabilitation and adaptive daily living training can also be created. In addition, remote consultations with allied health professionals such as dietitians, health psychologists and physiotherapists can be provided as an option for lifestyle management in dementia care.
7. **Enhance support systems for the ICs of PLWD.**
  A multifaceted approach needs to be implemented to address the persistent challenges faced by ICs. First, public education initiatives could be expanded by leveraging World Alzheimer’s Month events to increase public awareness. Targeted campaigns can also be developed to empower ICs about available resources and coping strategies. Second, caregiver-friendly workplace policies could be reinforced by encouraging flexible work arrangements for employees caring for PLWD. Third, a national framework for caregiver support should be established. Relevant ministries, local dementia associations, dementia care home providers and healthcare providers need to coordinate efforts to create a more comprehensive support network for ICs and PLWD. This can include leveraging telehealth technology to create virtual support groups with ICs. Lastly, a one-stop online information centre could be developed to provide knowledge about dementia, caregiving skills, directory and signposting information of local resources. At the time of writing, we are collaborating with the ADFM and World Health Organization to culturally adapt the ‘iSupport for Dementia’ web-based caregiver support programme into our national language.^50^ This web-based programme provides information on dementia, symptom management and self-care strategies for the ICs of PLWD in Malaysia. With this ongoing effort, we hope to set the ground for a psychosocial intervention that can be easily accessed by all stakeholders in supporting the ICs of PLWD across the nation.

## Conclusion

This paper describes the present gaps in dementia care for patients and their ICs in Malaysia. There is a dire need to increase the number of geriatric specialists in the nation, expand the primary care workforce and add community facilities to address the gaps in the national healthcare system. As ICs remain the main pillar of support for PLWD but are often the neglected ‘silent patients’, another crucial direction is to focus on the development of a culturally appropriate and easily accessible supportive intervention for them. This paper can guide policymakers and stakeholders in working together to address the urgent need for developing dementia care policies across the nation.
