# Corrigendum to "Caring for people living with dementia and their informal caregivers: Current perspectives in Malaysia"

**DOI:** 10.51866/cor.002

**Published:** 2025-01-26

**Authors:** Ken Joey Loh, Alvin Lai Oon Ng, Yook Chin Chia, Wan Ling Lee, Devi Mohan, Elil Renganathan

**Affiliations:** 1 DPsych (Clinical Psychology), FMASO, Department of Psychology, School of Medical & Life Sciences, Sunway University, Petaling Jaya, Selangor, Malaysia. Email: alvinn@sunway.edu.my; 2 MPsych (Clinical Psychology), Department of Medical Science, School of Medical & Life Sciences, Sunway University, Petaling Jaya, Selangor, Malaysia.; 3 MBBS, FRCP, Department of Medical Sciences, School of Medical & Life Sciences, Sunway University, Petaling Jaya, Selangor, Malaysia.; 4 Department of Primary Care, Medicine, Faculty of Medicine, University Malaya, Kuala Lumpur, Malaysia.; 5 Registered Nurse (RN), PhD (Nursing), Department of Nursing, Faculty of Medicine, Universiti Malaya, Kuala Lumpur, Malaysia.; 6 MBBS, MD (Community Medicine), GCHE, School of Public Health, The University of Queensland, Herston QLD 4006, Australia.; 7 MD, PhD, Jeffrey Cheah School of Medicine and Health Sciences, Monash University Malaysia, Jalan Lagoon Selatan, Bandar Sunway, Selangor, Malaysia.

In our article titled “Caring for people living with dementia and their informal caregivers: Current perspectives in Malaysia” published in this journal (Malaysian Family Physician (2024;19:69)),^1^ we would like to bring to the readers’ attention to certain discrepancies that have come to light.

The authors received feedback highlighting misreport of certain information in the aforementioned article. Specifically, on “Gap 2: Systemic challenges in primary care settings hinder the provision of comprehensive dementia care” (page 3), the sentences “Another commonly reported barrier is time constraints. At outpatient clinics in Malaysia, the average doctor-patient consultation time spans from 10 to 20 minutes. [21]” may be misleading. The original literature by Ahmad, Khairatul & Farnaza (2017)^2^ was not a nationwide study; rather, the study was conducted among the patients in one primary healthcare clinic in Malaysia. Hence, to clarify this misleading information, we have corrected the original sentences to: “Another commonly reported barrier is time constraints. A study conducted at a primary healthcare clinic in Malaysia revealed the average doctor-patient consultation time to span from 10 to 20 minutes. [21]”.

Another feedback on misreport was found in “Gap 4: The lack of awareness about dementia restricts help-seeking behaviour” (page 4). The sentence “Nonetheless, a recent cross-sectional study revealed that 92.8% of older adults in Malaysia, including those at risk of developing mild cognitive impairment, had low dementia awareness. [28]” might be misleading. The original literature by Ali, Ja’afar, Krishnan et al. (2023)^3^ was not a nationwide study; rather, the survey was conducted among the elderly patients in one university-based primary care clinic in Malaysia. Hence, this sentence has been corrected to: “Nonetheless, a recent cross-sectional study revealed that 92.8% of older adults from a university-based primary care clinic in Malaysia, including those at risk of developing mild cognitive impairment, had low dementia awareness.[28]”

We acknowledge the oversight in not explicitly clarifying the study populations of the respective publications in our article. We assure our readers that the rest of the information reported in our article remain accurate to the sources.
